# Genomic and phylogenomic analyses reveal extensive diversity among soil-derived *Acinetobacter baumannii* isolates from Nigeria

**DOI:** 10.1038/s41598-026-54394-3

**Published:** 2026-06-08

**Authors:** Ahmed Olowo-okere, Evelyn Skiebe, Gottfried Wilharm

**Affiliations:** 1 Department of Pharmaceutical Microbiology and Biotechnology, Faculty of Pharmaceutical Sciences, University of Abuja, PMB 117, Abuja, FCT Nigeria; 2 Project Group P2, Robert Koch Institute, Burgstr. 37, 38855 Wernigerode, Germany

**Keywords:** *Acinetobacter baumannii*, Population diversity, Ecology, Bacterial genomics, Nigerian environment, Genetics, Microbiology, Molecular biology

## Abstract

This study reports the genomic characterization of 24 representative *Acinetobacter baumannii* isolates recovered from soil across ecologically distinct sites in Nigeria’s Federal Capital Territory. From the 43 soil samples analyzed, 101 *Acinetobacter* spp. were recovered, predominantly *A. baumannii* (39/101; 38.61%). Analysis of the 24 *A. baumannii* isolates revealed extensive genetic diversity, including 20 distinct Pasteur and 19 Oxford sequence types (STs), of which 15 and 13, respectively, were novel. Additionally, we detected 20 capsular (KL) and seven outer core lipooligosaccharide (OCL) locus types. In silico prediction revealed that most isolates did not harbour clinically relevant antibiotic resistance genes and were unrelated to known international clonal complexes. However, one isolate (A23-4) belonged to the international clone 8 (IC8) lineage and harboured a class 1 integron-associated MDR cassette containing *ant(3*″*)*-Ia, *dfrA1*, and *sul2*, flanked by insertion sequences *ISAIw4*, *ISVsa3*, and *IS1006*. Further analyses identified 4,975 pangenome gene clusters and a polyphyletic distribution of the *A. baumannii* isolates across multiple distinct evolutionary lineages. The studied soil samples harbour a highly diverse and largely antimicrobial-susceptible population of *A. baumannii* rich in novel lineages. However, the detection of a MDR ST10/IC8 clone highlights the value of genomic surveillance in identifying potentially high-risk strains within environmental reservoirs.

**Supplementary Information:**

The online version contains supplementary material available at 10.1038/s41598-026-54394-3.

## Background

*Acinetobacter* species are a group of aerobic, non-sporulating, glucose non-fermentative, catalase-positive, oxidase-negative, Gram-negative coccobacilli^1^. Although classically defined as non-motile due to a lack of flagella, they exhibit complex surface-associated movement, including Type IV pili-mediated twitching and swarming motility^2^. As of March 2026, The genus currently comprises about 90 validly described species with standing in nomenclature^3^. *Acinetobacter baumannii* (*A. baumannii*), *A. nosocomialis* and *A. pittii* are the species most commonly implicated in varieties of clinical infections^4^. Around the world, numerous outbreaks of *A. baumannii* infections attributed to epidemic clones have been reported in hospitals, predominantly in critical care units^4^. The bacterium is renowned for its resistance to several antibiotic classes including the last resort antibiotics colistin and carbapenems^4^. This makes the management of *A. baumannii* infections highly challenging. Consequently, carbapenem-resistant *A. baumannii* (CRAb) has been designated as one of the critical priority pathogens by the World Health Organization (WHO)^5^.

The transformation of *A. baumannii* within a short span from a highly antibiotic susceptible into multidrug-resistant phenotypes is due to expansion of its genomic resistance islands as a result of antibiotic pressure within the hospitals in addition to its inherent natural antibiotic resistance^1^. This is further complicated by horizontal acquisition of antibiotic resistance genes through the promiscuous activity of mobile genetic elements^1^.

While members of the genus *Acinetobacter* are generally ubiquitous in nature, medically important members of the genus, the *A. baumannii*, were previously not considered ubiquitous^6^. This is especially true for multidrug-resistant (MDR) *A. baumannii* which have been isolated mostly from colonized or infected patients, hospital settings and anthropogenically impacted environments such as waste water^7^. Several attempts to isolate MDR *A. baumannii* from natural environment were unsuccessful^7^. For example, the attempt to isolate MDR *A. baumannii* from environment as a possible source of outbreak of MDR *A. baumannii* infections among United States service members injured in Iraq was unsuccessful^7^. The generally conspicuous absence of MDR *A. baumannii* in most pristine environment may be a result of loss of antibiotic resistance genes due to high fitness cost associated with its acquisition and maintenance in the absence of antibiotic pressure^8^. Conversely, the presence of MDR *A. baumannii* in anthropogenically impacted environments may suggest the existence of strong selective pressure, possibly from soil dwelling antibiotic producers, interacting competitively with *A. baumannii* or spilling of nosocomial strains into the environment^9,10^. Recent genomic evidence from Nigeria has demonstrated the bidirectional transmission of MDR *A. baumannii* between clinical settings and non-clinical environments, highlighting a porous interface between hospitals and the community^11^. Antibiotic secretion by antibiotic producing bacteria significantly changes environmental microbial diversity and composition. This has been shown to contribute to suppression of growth of susceptible bacteria, or cause surrounding bacteria cells to enter dormancy or become tolerant to the antibiotics, while other populations are inhibited^10^. These events could occur concurrently, culminating in shifts in microbial diversity. The population diversity could be further enriched by uptake of resistance genes from unmatched gene pools present in water, soil and other environments and exposure to man-made antibiotics^10^.

Beyond the clinical setting, it is increasingly well established that non-human *A. baumannii* isolates are remarkably ubiquitous and ecologically versatile. Globally, the bacterium has been recovered from a diverse array of natural sources and non-human hosts, including soil, flora, aquatic ecosystems, and various animal species^12–14^. While a number of studies have documented the isolation of clinical MDR *A. baumannii* in Nigeria^15–18^, data on genomic diversity and ecology of *A. baumannii* in Nigerian environment is highly limited. This study thus aimed to comprehensively characterise the genomic diversity, resistome, virulome, and global phylogenomic context of soil-derived *A. baumannii* isolates from Nigeria.

## Results

**Fig. 1:**
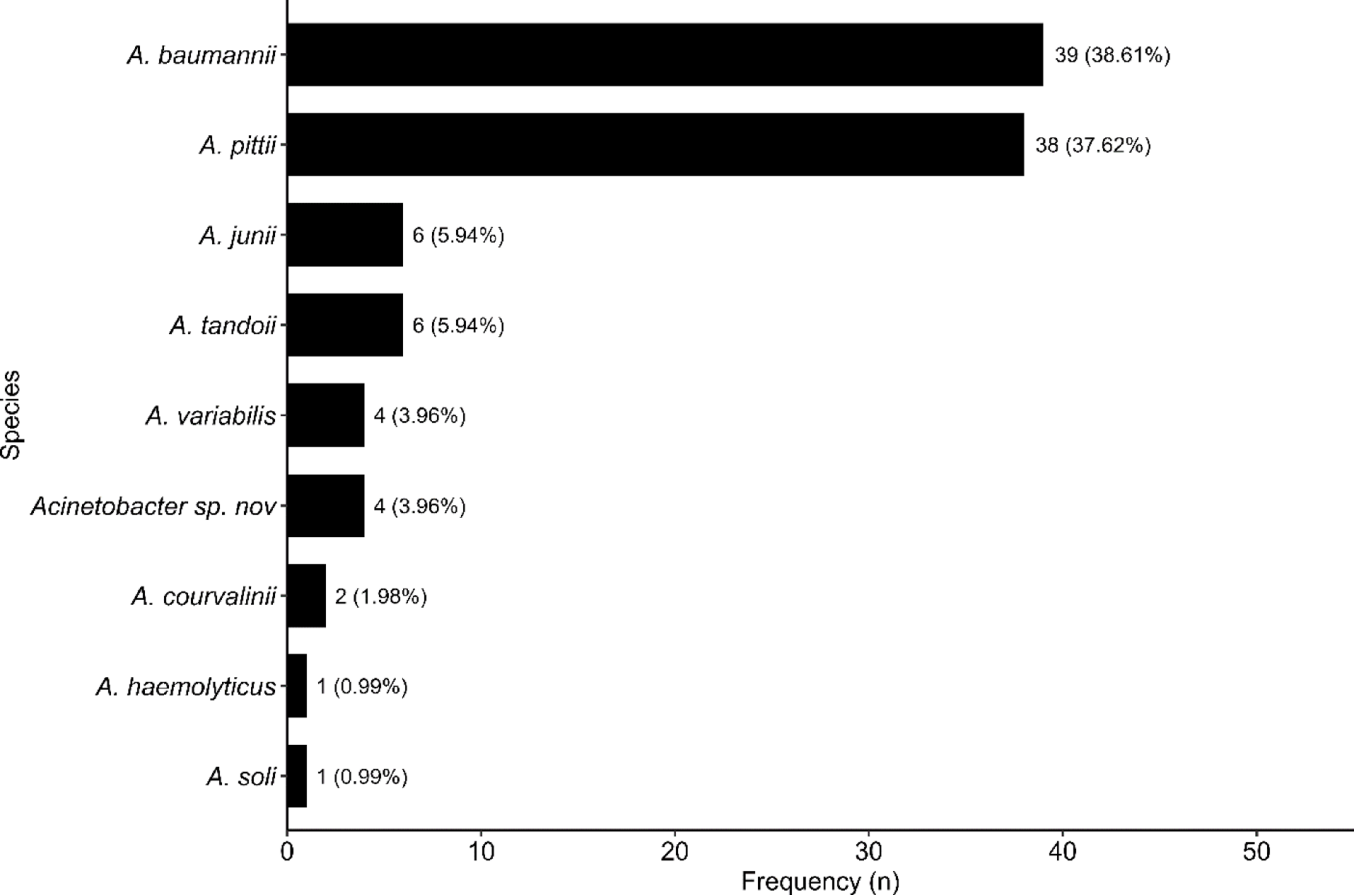
Distribution of Nigerian soil derived *Acinetobacter* species.

A total of 101 *Acinetobacter* spp. were isolated from the 43 soil samples studied, predominantly *A. baumannii* (*n* = 39; 38.2%), followed closely by *A. pittii* (*n* = 38; 37.3%). Additionally, 6 (5.9%) each of *A. tandoii* and *A. junii* were identified (Fig. 1). The isolated *A. baumannii* were recovered from 24 (55.8%) of the sampled sites. Interestingly, four isolates (Accession No: JBITPU000000000, JBIUCP000000000, JBIUCS000000000 & JBIUCQ000000000) could not be assigned to any known *Acinetobacter* species, as they exhibited < 96% ANI sequence similarity to all validly described *Acinetobacter* type strains.

### Genomic statistics and assembly quality

Assembly and quality statistics for the 24 studied bacterial genomes are summarized in Table S3. Genome completeness was consistently high, with 23 of the genomes exhibiting 100% completeness. Contamination levels were low across all assemblies, with isolate B14_2 having the maximum contamination value of 0.46%. The genome sizes ranged from 3.58 Mbp (A23-4) to 3.85 Mbp (A24_1). GC content was relatively uniform, ranging from 38.81% (C3_2) to 39.14% (B12-3). Median GC content of the genomes was 39.01%. N50 values spanned from 33,366 bp (B12-3) to 466,744 bp (C6-4). Assembly fragmentation, assessed using L50 and L90, ranged from 3 to 37 and 9 to 111, respectively (Table S3).

Prokka annotation summary revealed that the number of coding sequences (CDS) identified per genome ranged from 3,345 (A23-4) to 3,557 (C3-2), with gene counts between 3,497 and 3,625. The annotation also revealed between 3 and 8 *rRNA* genes, 61 to 64 *tRNA* genes, and a consistent presence of a single *tmRNA* gene across all genomes (Table S3). All the sequenced genomes have been deposited in the NCBI GenBank and their biosample and bioproject accession numbers are detailed in Table S4.

### MLST analysis

MLST analysis revealed that distinct genetically diverse *A. baumannii* populations exist in the studied soil samples. The 24 *A*. *baumannii* isolates belonged to 20 distinct Pasteur STs. Fifteen (*n* = 15, 75%) of which were novel STs (ST2810–ST2813, ST2815–ST2825). Other STs detected included ST10, ST280, ST309, and ST806. While ST806, ST2813, and ST2819 were each detected in multiple strains—three, two, and two strains respectively, the remaining STs were each found in single strains. Similarly, Oxford MLST analysis revealed a high degree of genetic diversity among the isolates. A total of 19 distinct STs were detected, of which 13 (68.4%), ST3518, ST3520–ST3526, and ST3528–ST3532, were novel. However, 1 strain (13.04%) could not be assigned to any defined ST because it lacks one typing locus.

**Fig. 2:**
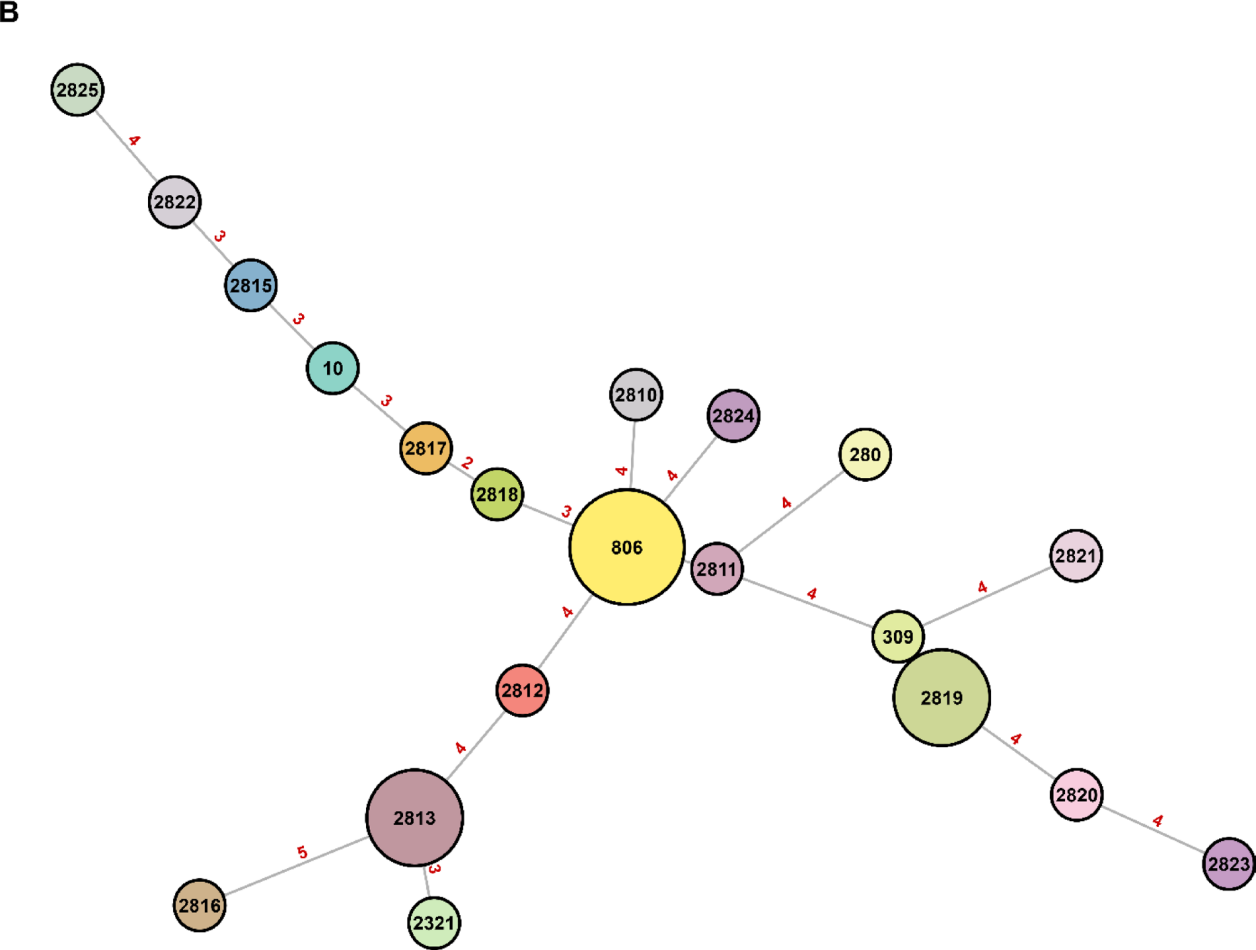
Minimum spanning tree (MST) depicting the population structure based on the standard 7-locus Pasteur MLST. Nodes (circles) represent unique Sequence Types (STs) identified within the studied genomes. The area of each node is proportional to the number of genomes assigned to that ST, with larger nodes indicating higher-frequency clones. Nodes are colored arbitrarily to distinguish unique profiles. Branches connecting the nodes represent the minimum genetic distance, labelled in red with the absolute number of allelic differences (Hamming distance) between linked Sequence Types.

The relatedness of the genomes based on the Pasteur MLST scheme is presented in Fig. 2 and additional details on the allelic profiles identified in each of the genomes is available in Table S5. The minimum spanning tree revealed substantial genetic heterogeneity across the dataset, with most isolates separated by 3 to 5 allelic mismatches. Interestingly, most of the isolates (23/24, 95.2%) belonged to none of the twelve known ICs. One of the isolates, identified as ST10, however is a member of international clone 8 (IC8).

**Fig. 3:**
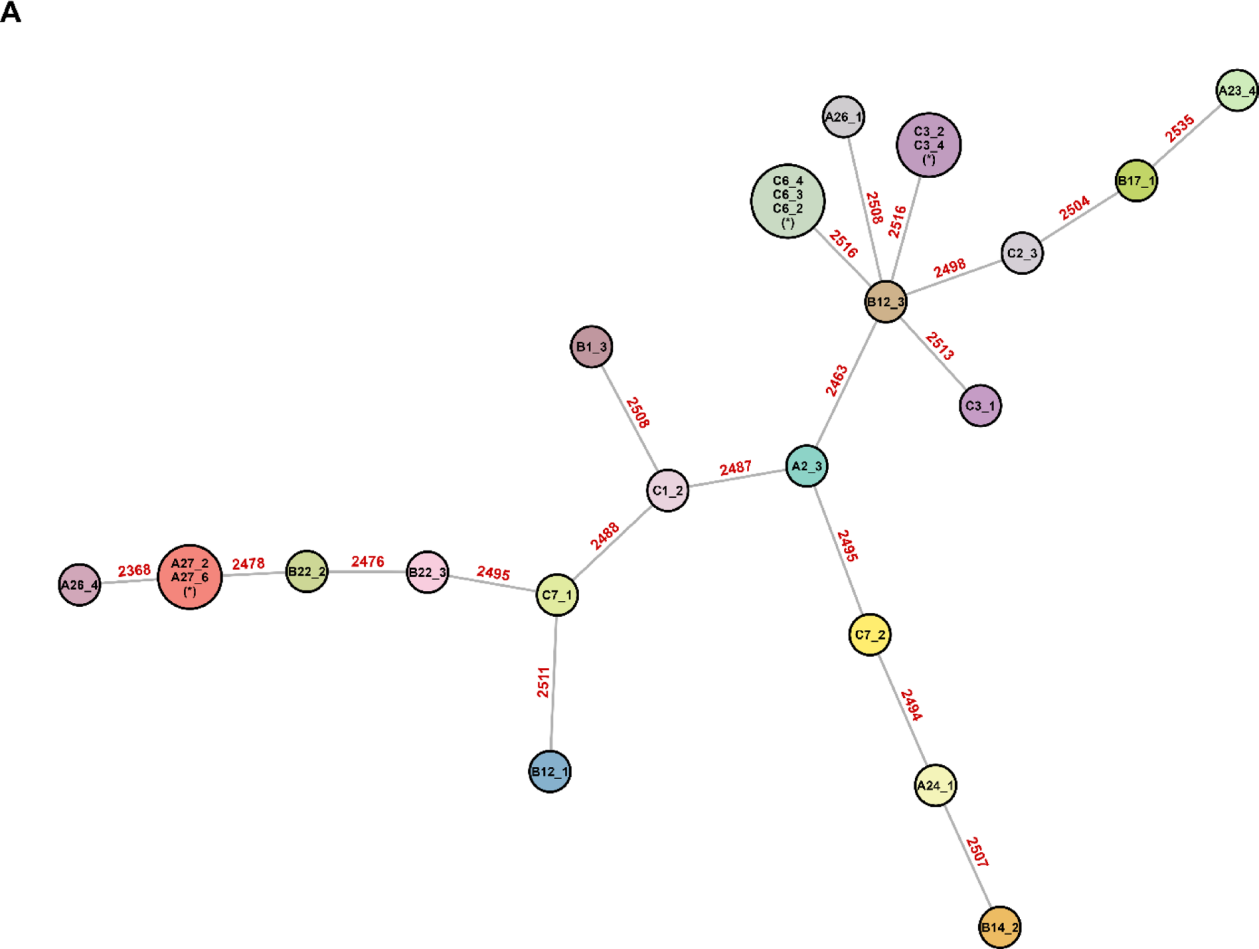
Minimum spanning tree (MST) depicting the high-resolution population structure based on Core Genome MLST (cgMLST). Nodes (circles) represent core genome allelic profiles based on 95% core loci. Branches connecting the nodes represent the minimum genetic distance, labelled in red with the absolute number of allelic differences (Hamming distance) between linked profiles. To improve visualization of the broader population structure, closely related clonal complexes exhibiting ≤ 15 allelic differences were collapsed into representative meta-nodes (scaled proportionally by size and indicated by an asterisk). Specifically, the collapsed clonal groups and their internal pairwise allelic differences are as follows: [C3_2, C3_4 (10 alleles)]; [A27_2, A27_6 (8 alleles)]; [C6_4, C6_3, C6_2 (2–13 alleles)].

The cgMLST analysis (Fig. 3) revealed a highly diverse population structure characterized by long branch lengths between most isolates. The majority of the connected nodes exhibited large allelic distances, typically exceeding 2,000 allelic differences (range: 2,396 to 2,566), indicating that most isolates are genetically distinct and phylogenetically distant. However, the high discriminatory power of cgMLST also identified a few small, closely related clusters. Notably, isolates A27-2 and A27-6 formed a tight pair separated by only 8 allelic differences, suggesting a recent common ancestor or a direct clonal relationship. Similarly, small clusters were observed for isolates C3-2 and C3_4 (10 allelic differences) and between C6_2, C6_3 and C6-4 (2–13 allelic differences).

### Antimicrobial resistance gene, IS elements and plasmid typing distribution

**Fig. 4:**
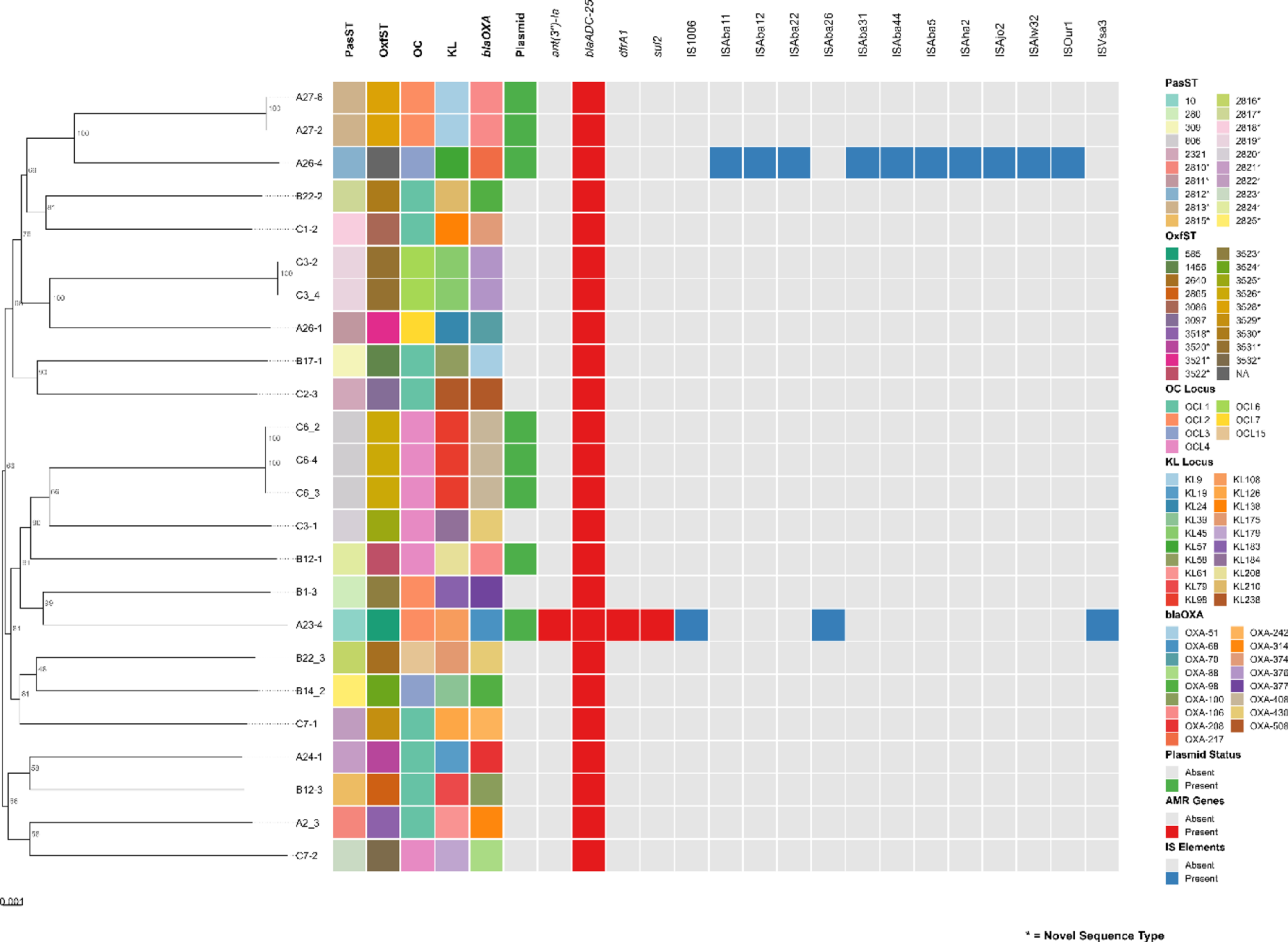
Phylogenetic tree and genomic profiles of the studied *A. baumannii* isolates. The maximum-likelihood phylogeny illustrates the evolutionary relationships among the 24 *A. baumannii* isolates, with branch lengths representing true genetic distances. Bootstrap support values (percentages) are provided at the internal nodes. The adjacent grid to the right of the tree details the genomic characteristics of each strain, aligned with the tree topology. Columns denote Sequence Types (ST) determined via the Pasteur (PasST) and Oxford (OxfST) MLST schemes, outer core (OC) and capsular polysaccharide (KL) loci, and specific *bla*_OXA−51_-like gene variants. The binary heatmaps indicate the presence (colored) or absence (light grey) of detected plasmids (green), antimicrobial resistance (AMR) genes (red), and insertion sequence (IS) elements (blue). Asterisks (*) indicate novel sequence types and NA, not assigned.

**Fig. 5:**
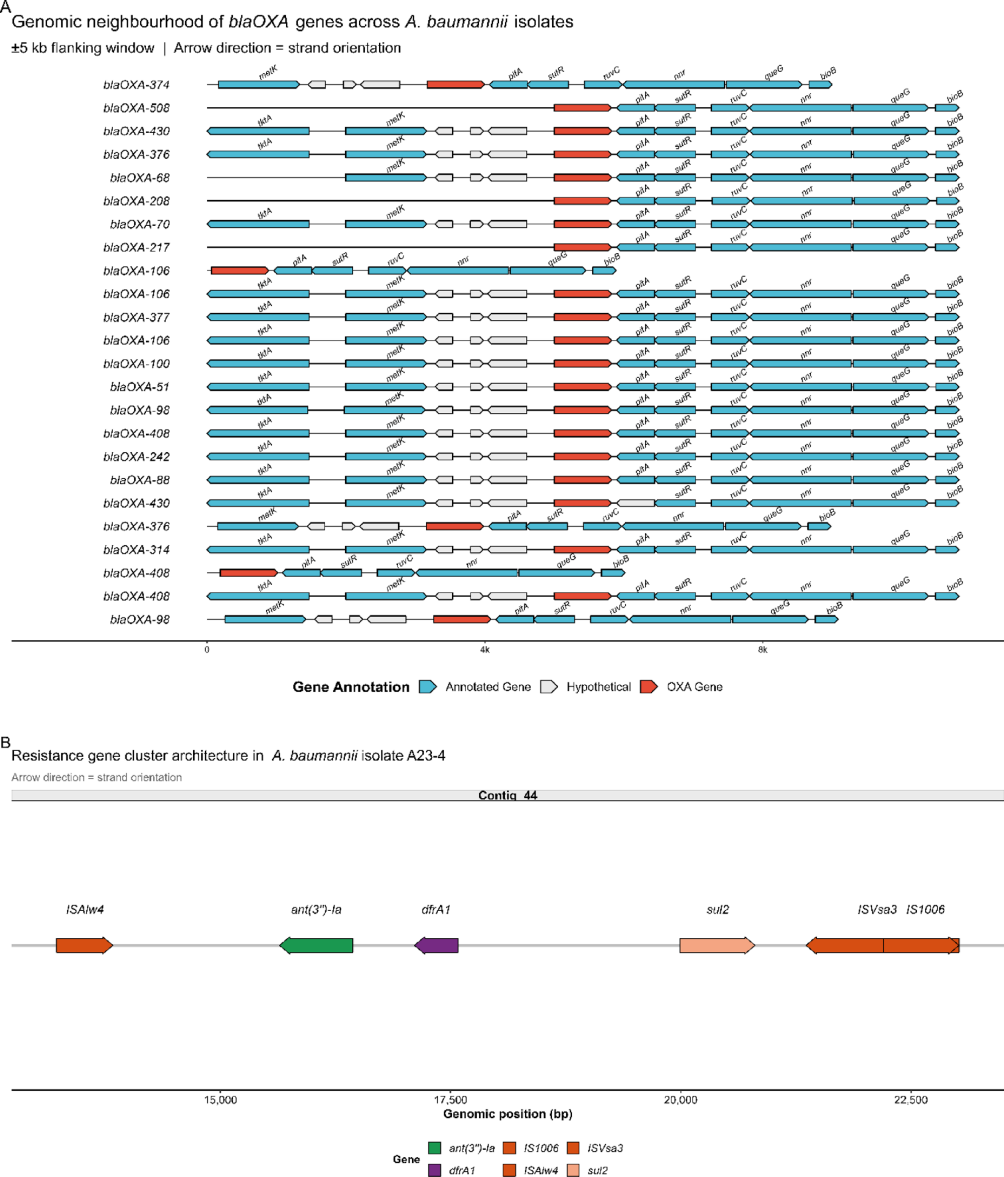
Genomic context of *bla* OXA genes and flanking mobile genetic elements in the studied *A. baumannii* genomes. Panel (**A**) Genomic neighbourhood of *bla*OXA genes across *A. baumannii* isolates. The genomic context surrounding *bla*OXA loci is shown for each sequenced isolate within a ± 5 kb flanking window. Red arrows denote identified OXA β-lactamase genes; blue arrows represent annotated functional genes; grey arrows indicate hypothetical proteins. Arrow length is proportional to gene size, and arrow direction indicates strand orientation of transcription. Panel (**B**) Resistance gene cluster architecture in *A. baumannii* isolate A23-4. Detailed gene map of Contig 44 from isolate A23-4, illustrating a class 1 integron-associated multidrug resistance (MDR) gene cluster. Arrow length is proportional to gene size, and arrow direction indicates strand orientation.

Overall, AMR prediction using the ResFinder database showed that the isolates harboured between 2 and 5 AMR genes per genome. The majority of isolates (23/24; 95.8%) harboured only two (2) resistance genes, with only strain A23-4 harbouring 5 AMR genes. While all isolates harboured the intrinsic *bla*_OXA−51_-like gene, their variants differed considerably, with 17 distinct variants identified; The most prevalent variants were *bla*_OXA−106_ and *bla*_OXA−408_, present in 12.5% (3/24) of isolates each. The remaining variants (*bla*_OXA−100_, *bla*_OXA−208_, *bla*_OXA−242_, *bla*_OXA−314_, *bla*_OXA−374,_ *bla*_OXA−376,_ *bla*_OXA−377,_ *bla*_OXA−430,_ *bla*_OXA−70 and_ *bla*_OXA−88_) were each found in 1 to 2 isolates (Fig. 4). Details of other genes, identified using the CARD RGI tool, including disinfectant resistance gene (*qacG*), global regulatory elements (*adeR*,* adeS*,* adeN*,* adeL*), and multidrug efflux systems (*AdeABC*,* AdeFGH*,* AdeIJK*,* AbaF*,* AbaQ*,* AmvA*,* abeM*,* abeS*), are presented in Table S6. Analysis of the genomic context surrounding the *bla*_OXA_ genes (± 5 kb) confirmed a complete absence of insertion sequence (IS) elements, such as *ISAba1*, either upstream or downstream of the *bla*_OXA_ genes across all analyzed isolates. Synteny analysis revealed that the *bla*_OXA−51_-like genes are situated within a highly conserved chromosomal environment, characteristically flanked upstream by *pitA* and *sutR* (Fig. 5A).

In stark contrast to the stable intrinsic loci surrounding the *blaOXA* regions, analysis of the genetic environment flanking the *ant(3*″*)*-Ia gene in isolate A23-4 revealed a clustered multidrug resistance (MDR) region bounded by mobile genetic elements. This mobilizable cassette, consistent with a class 1 integron-associated structure, harboured the aminoglycoside resistance gene *ant(3*″*)*-Ia, the trimethoprim resistance determinant *dfrA1*, and the sulfonamide resistance gene *sul2*. The cassette was flanked upstream by the insertion sequence *ISAIw4* and downstream by *ISVsa3* and *IS1006*, a configuration strongly suggestive of IS-mediated acquisition and horizontal dissemination of this resistance region (Fig. 5B).

Plasmid replicon typing revealed that only 8 (33.3%) of the analysed isolates harboured at least one plasmid. The most frequently detected replicon was pABLAC1, which was present in six isolates (A23-4, A27-2, A27-6, C6-4, C6_3, and C6_2). The pB8300 plasmid was also identified in two isolates (A26-4 and B12-1). Notably, co-carriage of multiple plasmids was observed in three isolates: A23-4 harboured both pABLAC1 and pKCRI-43-1; A26-4 co-carried pB8300 and p2FDAARGOS_533; and B12-1 contained both pB8300 and pABA-6973 (Table S7).

### Pangenome analysis

The pangenome analysis revealed a total of 4,975 gene clusters, comprising 2,671 core genes, 196 soft-core, 958 shell, and 1,150 cloud genes (Table S8).

### Outer polysaccharide capsular (KL), lipooligosaccharide outer core (OCL) locus types and virulence genes in *Acinetobacter* genomes

The KL and OCL locus types analysis revealed high diversity in KL types, with 20 distinct KL types identified, including KL9, KL19, KL24, and KL98 appearing in multiple isolates. In contrast, fewer OCL locus types were observed, with OCL1 and OCL4 being the most common (Fig. 4).

### Virulence determinants

The result of virulence genes prediction revealed the presence of a wide array of virulence genes in the 24 genomes analysed. Five virulence genes (*clpP*, *htpB*, *kdsA*, *manB*, and *pilT*) were found to be conservatively present in all 24 *A. baumannii* genomes (Table S9). Others such as *ureB* and *tviB* were found in most (20–22) genomes, and *cap8E*, *rffG*, *bplB*, and *galU* were rarely detected.

### Phylogenomic reconstruction and global contextualization

**Fig. 6:**
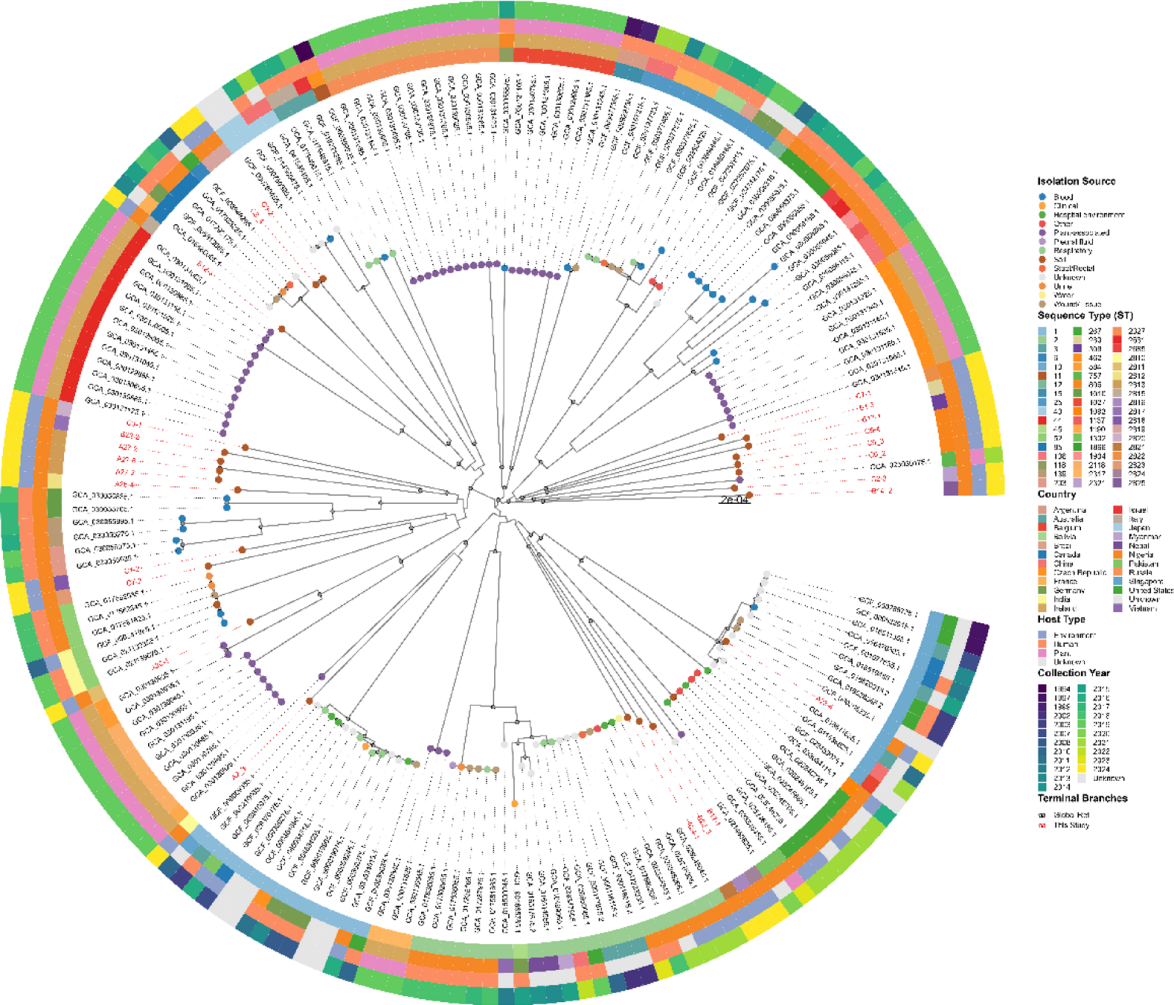
Maximum-likelihood Phylogenetic analysis of *A. baumannii* isolates from this study alongside publicly available genomes from Nigeria and international reference genomes. The maximum likelihood phylogenetic tree was constructed from a core genome alignment of 24 *A. baumannii* isolates sequenced in this study (highlighted in red), all publicly available *A. baumannii* genomes from Nigeria, and representative genomes from major international clonal complexes (ICCs). The tree is rooted at the midpoint. The scale bar at the centre represents the number of nucleotide substitutions per site. Grey nodes indicate bootstrap support values > = 70%. Tips with text coloured in red represent isolates sequenced in this study, while black text denotes global reference genomes. Coloured dots at the branch tips indicate the specific isolation source. Concentric rings mapping isolate metadata (from innermost to outermost) represent: (1) Sequence Type (ST), (2) Country of origin, (3) Host type, and (4) Collection year. Missing or unknown metadata is represented by grey tiles.

Phylogenetic reconstruction of the study isolates against a diverse array of global genomes revealed a polyphyletic distribution, with our sequenced *A. baumannii* isolates dispersing across multiple distinct evolutionary lineages (Fig. 6). This topological scattering indicates a high degree of genetic diversity, consistent with our earlier MLST results that showed that the isolates are genetically distinct.

The isolate A23-4 congregated firmly within the well-supported ST10 subclade. The phylogram places this isolate in close proximity to GCA_016511635.1. Genomic analysis confirmed significant conservation between A23-4 and GCA_016511635.1, with a core genome divergence of only 64 SNPs, confirming they belong to the same distinct genomic lineage and a close evolutionary relationship (Table S10).

A co-clustering of Nigerian soil isolates from the present study with previously sequenced Nigerian *A. baumannii* genomes was observed, with a distinct subset of the study isolates, including C3-1, B22-2, A27-2, and A27-6, forming a well-supported monophyletic subclade alongside several genomes of Nigerian origin.

## Discussion

This study provides one of the first genomic characterizations of soil derived *A. baumannii* isolates from Nigeria. It reveals extensive species diversity, sequence types including several novel STs, and a repertoire of AMR genes and capsular polysaccharides. This study enhances our understanding of ecological adaptation and diversity of *A. baumannii* in Nigeria.

While the isolation of *A. baumannii* from environmental sources including hospital waste water and fresh water has been previously reported^16,19,20^, this study represents one of the few documented isolation of *A. baumannii* from soil samples in Nigeria. Recently, a study described the isolation of *A. baumannii* isolates from both hospital and non-clinical settings in northern Nigeria^11^. Similar to the findings of this study, *A. baumannii* has been isolated from diverse environmental contexts such as freshwater, wastewater, and animal production environments in South Africa and India^21,22^.

The isolation rate of 55.8% observed in this study (24 out of 43 positive samples), is markedly higher than the ~ 30% prevalence reported in a large ecological study of environmental *A. baumannii* in Europe^23^. The relative abundance and widespread distribution of *A. baumannii* in the study area compared to Europe may be due to differences in local selective factors such as antibiotic use, climate, or human–animal–environment interactions. Previous studies have shown that *A. baumannii* prevalence correlates with higher ambient temperatures, which can enhance resistance gene transfer and environmental persistence^24^. Furthermore, limited infection control, empirical and over the counter antibiotic use, and the presence of environmental reservoirs such as abattoirs and contaminated water sources in some LMICs have been implicated in sustaining local transmission^25–28^. These ecological and anthropogenic factors create conditions conducive to the proliferation and dissemination of multidrug-resistant *A. baumannii*.

The isolated *A. baumannii* exhibited distinctive genomic diversity. The detection of 15 novel Pasteur STs in this study may indicate distinctiveness of the *A. baumannii* populations in the study area. This is in line with an earlier finding from Nigeria, where studies of clinical *A. baumannii* isolates identified a range of unique Oxford STs^19,20^. Our detection of ST 10 (IC8) in a soil isolate raises the concerning possibility that such high-risk clones may circulate or persist in environmental reservoirs outside healthcare settings. This is particularly alarming given that ST10 includes hypervirulent *A. baumannii* LAC-4 strain, which is associated with increased virulence and transmissibility^29^. In Nigeria, IC8 lineage has already been established as important clinical threats. For instance, several studies have confirmed its widespread circulation, more concerning among *bla*_OXA−23_ bearing carbapenem resistant *A. baumannii* in hospitals across multiple geopolitical zones^19,20^. The detection of this lineage in our soil sample establishes Nigerian soil not merely as an incidental sink, but as a clinically relevant community reservoir. This is further complicated by AMR potential of the isolates. Notably, isolate A23-4 carrying *dfrA1*,* sul2* and *ant(3*″*)*-Ia on mobilizable resistance islands may pose a potential public health threat by facilitating the horizontal transfer of acquired resistance determinants to clinically relevant isolates in their immediate environment. This therefore underscores the urgent need for integrated One Health genomic surveillance in resource-limited settings to monitor and mitigate the spread of multidrug-resistant pathogens outside the hospital ecosystem to inform effective infection control strategies. It should be noted that the resistance profiles reported in this study are based solely on in silico predictions derived from genomic data. While the presence of resistance-conferring genes is a strong indicator of a resistant genotype, phenotypic expression may vary.

Pangenome and phylogenomic analyses revealed distinct lineages and genetic signatures within the studied genomes. Localized selective pressure may account for the divergent evolutionary dynamics observed among the studied isolates, as evidenced by the heterogeneous distribution of the intrinsic *bla*_OXA_ gene variants. These findings align with studies from Nigeria and other African countries, where *A. baumannii* isolates exhibit high genetic diversity and a broad spectrum of resistance genes. In southwestern Nigeria, for instance, isolates displayed diverse sequence types and harbored carbapenem resistance genes^16,19^.

This study is not without limitations. First, collecting samples exclusively from the Federal Capital Territory limits the generalization of the findings to the broader environmental *A. baumannii* population across Nigeria. Second, phenotypic antimicrobial susceptibility testing was not performed, thereby limiting direct correlation between genotypic and phenotypic resistance. Third, reliance on short-read sequencing may have hindered the detection of certain genomic features, such as mobile genetic elements and virulence genes, which are better resolved with long-read sequencing technologies. Lastly, the lack of metadata on soil composition and antibiotic contamination prevented a comprehensive analysis of environmental factors influencing the observed genomic diversity.

Despite these limitations, this study provides a first glimpse into the genomic features of soil derived *A. baumannii* in Nigeria. Future studies should incorporate long-read sequencing to resolve complete chromosome and plasmid architectures, functional validation of predicted resistance and virulence genes, and phenotypic antimicrobial susceptibility testing to bridge the gap between genomic predictions and clinical relevance.

## Conclusion

This study demonstrates that Nigerian soil harbours genetically diverse, mostly antibiotic susceptible *A. baumannii* isolates. The detection of a high-risk clone (ST10/IC8) isolate bearing an array of acquired antibiotic resistance genes highlights the importance of environmental surveillance and the integration of One Health approaches in AMR monitoring strategies, particularly in regions facing increasing antibiotic pressure and limited clinical diagnostic capacity.

## Methods

### Ethical approval and permission to collect samples

This study did not involve human participants, animals, endangered or protected species, therefore, ethical approval and informed consent were not required. However, official authorization for sample collection for this study was obtained from the National Health Research Ethics Committee (NHREC), Federal Ministry of Health, Abuja, Nigeria (NHREC/032/05/2024).

### Description of study areas

Collection of soil samples was carried out from two area councils in Nigeria’s Federal Capital Territory: Gwagwalada area council and Abuja Municipal Area Council, Nigeria, in August 2024. Gwagwalada is one of the six area councils in Abuja, serving as a major educational and commercial hub, home to the University of Abuja and several government institutions. Iddo is a community within Abuja Municipal Area Council (AMAC), located along Airport Road, known for its growing residential and agricultural activities.

### Sample collection

Approximately 30 g of soil samples were collected from a depth of about 20 cm using a sterile spatula. A separate sterile spatula and glove were used for each sampling site. Detailed descriptions of the sample collection sites and their geographical coordinates are provided in Table S1. Sampling locations comprised diverse non-clinical environments across Abuja, Nigeria, including river sediments and soil from rock crevices. These distinct ecological niches were selected to investigate the extent to which *A. baumannii* colonizes the natural environment. This approach provides critical insights into the genomic diversity of the pathogen outside of healthcare facilities, helping to define its role as an environmental reservoir in urban settings. The sampling date and spatial variables for each site were recorded.

### Culture, isolation, and identification of *Acinetobacter* spp.

The collected samples were selectively enriched for *Acinetobacter* species using a previously published protocol^30^. Briefly, soil samples (1 g each) were enriched in 5 mL of mineral salt medium supplemented with 0.2% sodium acetate, vortexed for 30 s, and incubated at 37 °C for 5 h with shaking (100 rpm). A 100 µL aliquot of the broth was plated onto CHROMagar™ Acinetobacter without the multidrug-resistant (MDR) supplement and incubated at 37 °C for 24 h. Presumptive *A. baumannii* colonies (smooth, circular, pink to reddish) were sub-cultured onto Columbia agar supplemented with 5% sheep blood for purification and stored in 40% glycerol at − 80 °C.

### Molecular identification

The identity of the presumptive *A. baumannii* was determined by the amplification and sequencing of *A. baumannii* intrinsic *bla*_OXA−51−like_ gene following a previously published protocol^31^. The resulting sequences were compared against the NCBI non-redundant nucleotide database using the BLASTn algorithm^32^. Isolates that tested negative for the *bla*_OXA−51−like_ gene were subsequently identified to the species level via the amplification and sequencing of the RNA polymerase beta subunit (*rpoB*) gene, followed by BLASTn analysis as described previously^33^.

### Whole-genome sequencing and analysis

A representative subset of 24 morphological distinct isolates was selected from the 39 isolated *A. baumannii* isolates for genomic analysis. Genomic DNA was extracted from 24 randomly selected *A. baumannii* isolates using the MasterPure DNA Purification Kit (Epicentre Technologies, Madison, WI) and quantified with a Qubit 4.0 Fluorometer (Thermo Fisher Scientific) using the dsDNA High Sensitivity (HS) Assay Kit (Invitrogen), according to the manufacturers’ instruction. Libraries were prepared with the Illumina Nextera XT DNA Library Preparation Kit (Illumina, San Diego, CA, United States). and sequenced on an Illumina NextSeq platform (Illumina, San Diego, CA, United States) with a 2 × 150 paired-end configuration, generating high-depth coverage. Paired-end reads were processed and assembled using Shovill v1.1.0 (https://github.com/tseemann/shovill), an integrated wrapper pipeline. Within Shovill, raw reads underwent adapter removal and quality trimming via Trimmomatic v0.40, followed by assembly using SPAdes v4.2.0 as the core assembler, and subsequent error-correction and polishing, all performed internally within Shovill, to produce a high-quality final assembly^34,35^. Genomes lower than 3.5 Mb or larger than 4.2 Mb in size, or N50 scores < 25,000 and contamination level exceeding 5% were excluded from downstream analysis. The quality metrics of the assembled genomes, completeness and contamination were assessed using QUAST v 5.2.0 and CheckM2 v1.0.2^36,37^.

The species identity was initially done using bactinspectior v0.1.3 and further confirmed via pairwise average nucleotide identity (ANI) comparison using FastANI v 1.33 against the *A. baumannii* ATCC 19,606^T^ (ASM1933165v1)^38,39^. As previously described, any isolates with ANI value ≥ 96% were considered accurately identified^40^. Genome annotation was done using Prokka v1.14.6^41^ and multi-locus sequence typing (MLST) was performed with the MLST tool v2.23.0 (https://github.com/tseemann/mlst)^42^ using both the Pasteur and Oxford schemes. Genomes that could not be typed were tentatively considered novel ST and their allelic profiles were extracted and submitted to the PubMLST database (https://pubmlst.org) for ST assignment. A minimum spanning tree (MST) based on allelic differences among the isolates was constructed using the MSTree V2 algorithm^43^. Furthermore, core genome MLST (cgMLST) inference was performed using chewBBACA v3.5.1^44^. The core genome, defined as loci present in at least 95% of the isolates, was extracted and visualized as a minimum spanning tree.

### Antibiotic resistance gene, plasmid and insertion sequence elements determination

The assembled genomes were rapidly screened for antibiotic resistance genes using ABRicate v1.0.1 (https://github.com/tseemann/abricate) against the ResFinder database accessed March 2026^45,46^. The analysis was performed using default minimum coverage threshold of ≥ 80% and a minimum sequence identity of ≥ 80%. The presence or absence of resistance genes was visualized as a heat map and annotated onto the core genome phylogenetic tree. To further characterize the repertoire of AMR genes, the isolates were screened against the Comprehensive Antibiotic Resistance Database (CARD) using the Resistance Gene Identifier (RGI) tool with default parameters^47^.

To determine the genomic context of the *bla*_OXA_ genes, a 10-kb flanking region was defined for each locus, comprising the *bla*_OXA_ coding sequence and 5-kb of upstream and downstream flanking sequences. Gene features within each window were extracted, and visualized using gggenomes v 1.1.3^48^.

Insertion sequences and plasmid replicons were identified using ABRicate v1.0.1 against a custom ISfinder database (5,970 sequences, https://isfinder.biotoul.fr, accessed March 2026))^49^ and the *Acinetobacter* plasmid typing (ABAplasmids) database (152 sequences, accessed March 2026)^50^ respectively, with the default minimum nucleotide identity and coverage thresholds of 80%.

### Virulence genes, capsular polysaccharide (KL) and lipooligosaccharide outer core (OCL) detection

Genome assemblies were screened using ABRicate v1.0.1 against the virulence factor database^51^ with a less stringent thresholds set at ≥ 60% nucleotide identity and ≥ 60% query coverage. Resulting ABRicate outputs were summarized using the abricate—summary function. Kaptive v2.0.5 was employed to characterize capsular polysaccharide (KL) and lipooligosaccharide outer core (OCL) using the *A. baumannii* K locus and OC locus reference databases^52^. Only loci with at least good confidence match were reported.

### Pan-genome and phylogenetic analysis

The pan-genome, the collective set of genes present in the analysed genomes, was calculated using Panaroo v 1.6 with –strict option^53^. The genes were categorized as core (present in ≥ 99% isolates), soft-core (95–99%), shell (15–95%), and cloud (< 15%) genes. The core-genome alignment from Panaroo was processed with SNP-sites v2.5.1 to extract polymorphic sites, and pairwise SNP distances were computed using snp-dists v1.2.0^54^^,55^. The resulting SNP alignment was used to construct a maximum-likelihood phylogeny with IQ-TREE v2.2.0 with ModelFinder Plus for automatic model selection, constant site correction, and branch support assessed by 1,000 UFBoot2 and SH-aLRT replicates ^56^. The midpoint-rooted phylogenetic tree was visualized and annotated using ggtree v3.16.3 and ggtreeExtra v1.18.1 ^57^^,58^.

To further investigate the evolutionary placement of our genomes in the context of global *A. baumannii* diversity, we downloaded representative genomes from each of the recognized international clonal lineages, as well as all publicly available *A. baumannii* genomes from Nigeria ^59^ (Table S2). A core genome phylogeny was then constructed as described above.

### Statistical analysis

Descriptive statistics, including frequencies and percentages, were used to summarize categorical variables across isolates. All statistical analyses and visualizations were performed in R v4.5.1 using RStudio v2024.12.0, with figures generated using ggplot2 v4.0.1.

## Supplementary Information

Below is the link to the electronic supplementary material.

Supplementary Material 1

## Data Availability

The genome sequence data generated and analyzed during the current study are available in the National Center for Biotechnology Information (NCBI)’s Genomes database under BioProject ID: PRJNA1183969. The genome accession and BioSample numbers for each sample are listed in Table S4 in the supplemental material. The genomes of other isolates used in constructing the phylogenetic tree and comparative genomic analysis were retrieved from NCBI (https://www.ncbi.nlm.nih.gov/) and presented in Table S2. All supporting data, has been provided within the article or through supplementary data files.
